# Supplementation of specific insoluble fibres partially attenuates compromised growth performance of broilers fed wheat-based reduced crude protein diets

**DOI:** 10.1016/j.aninu.2025.06.009

**Published:** 2025-08-26

**Authors:** Nishchal K. Sharma, Sarbast K. Kheravii, Mingan Choct, Karen Gurney, Shu-Biao Wu

**Affiliations:** a School of Environmental and Rural Science, University of New England, Armidale 2350, NSW, Australia; b Redsun Nutrition Pty Ltd., Munruben 4125, QLD, Australia

**Keywords:** Broiler, Insoluble fibre, Performance, Reduced protein diet, Soy hull, Sugarcane bagasse

## Abstract

Insoluble fibre has been shown to be a functional fibre that helps the effective use of nutrients and energy through the gastrointestinal tract, and as such may be added to diets to enhance the growth performance of broilers. This study aimed to investigate the effects of insoluble fibre sources in a reduced protein (RP) diet fed to broilers. At d 10 post–hatch, 672 d-old Ross 308 male birds were assigned to 6 treatments with 8 replicates of 14 birds each. The treatments were a normal protein (NP) diet, a RP diet (−20 g/kg protein), and RP diets formulated with either sugarcane bagasse at 20 g/kg, lignocellulose-based fibre at 10 g/kg, oat hulls at 30 g/kg, or soy hulls at 30 g/kg. The results showed that the reduction in dietary protein decreased feed intake and weight gain, and worsened feed conversion ratio (FCR; *P* < 0.05). The birds fed the RP diet with soy hulls or bagasse had a better FCR (*P* < 0.05) compared to those fed the RP diet without fibre and similar FCR to those fed a NP diet. Bagasse or soy hulls supplementation in the RP diet decreased (*P* < 0.05) the starch digestibility coefficient compared to the RP diet without fibre. Lower counts (*P* < 0.05) of *Lactobacillus* in birds offered the RP diet with bagasse or oat hulls was observed compared to the RP diet alone. The difference (*P* = 0.011) in the expression of digestive enzyme genes in response to dietary treatments was observed for the *AMY2A* gene in the pancreas. The reduction in dietary crude protein (CP) downregulated (*P* < 0.05) the *AMY2A* gene and the addition of the four insoluble fibres to the RP diet had no effect on the expression of *AMY2A* compared to the RP treatment but bagasse, lignocellulose, and soy hulls added RP diets had similar *AMY2A* expression as the NP treatment. It was concluded that reducing dietary protein compromised the growth performance of broilers which was partially attenuated by supplementing the diets with bagasse or soy hulls.

## 1 Introduction

The benefits and challenges of feeding wheat-based reduced protein (RP) diets to broilers have been well documented (Greenhalgh et al., 2020; Selle et al., 2022; Sharma et al., 2017). Some of the benefits reported in the above studies include the reduced environmental impact of poultry production through improved litter quality, lower nitrogen excretion, lower ammonia and odorant emissions, improved bird health and welfare, and reduced dependence on imported soybean meal. The challenges include compromised growth performance and increased body fat and fat pad weight. Broiler performance is compromised at various magnitudes when dietary crude protein (CP) is reduced in wheat-based diets characterized by lower feed intake (FI) (Greenhalgh et al., 2020), lower weight gain (WG), greater feed conversion ratio (FCR), and higher abdominal fat pad weight (Chrystal et al., 2021; Macelline et al., 2023,b; c). Thus, nutritional strategies to improve the performance of broilers offered RP wheat-based diets have attracted interest in the industry, and various approaches have been explored including the use of enzymes and the supplementation of non-essential amino acids (AA) (Cowieson et al., 2017; Dean et al., 2006; Selle et al., 2024).

Broiler diets lack structural components which results in poor gizzard development and digestive function (Mateos et al., 2012; Svihus, 2011). In this study, insoluble fibre was chosen as a dietary ingredient to provide benefits to a RP feeding program as insoluble fibres provide a structural component to feed, and have been shown to improve gizzard function, nutrient digestibility, and growth performance in broilers (Jimenez-Moreno et al., 2013; Kheravii et al., 2017; Mateos et al., 2012). As an example, WG and FCR of broilers were improved when oat hulls or soy hulls were included in the diet at 30 g/kg (Barekatain et al., 2017; Gonzalez-Alvarado et al., 2007; Jimenez-Moreno et al., 2013). Similarly, in another study, supplementation of 20 g/kg sugarcane bagasse over the top of a standard corn-soybean based broiler diet improved CP digestibility by 3% (Kheravii et al., 2017). Sugarcane bagasse, oat hulls, soy hulls, and lignocellulosic biomass are all insoluble fibre sources as they contain high levels of insoluble non-starch polysaccharides (NSP). Adding insoluble fibre in RP diets possibly conserves the benefits of post-pelleted whole grain addition as currently practiced by the industry, such as increased gizzard size and function, but also prevents the selective intake of whole grains by birds, resulting in the balanced intake of nutrients, presumably lower intestinal viscosity (due to lower wheat inclusion) and effective regulation of the flow of nutrients in the gut. A RP wheat-based diet has increased levels of rapidly digestible starch coming from wheat compared to a normal protein (NP) diet (Chrystal et al., 2021), so pellet feed formulated with insoluble fibre may be a better option when dietary CP is reduced as this will: a) increase the dietary level of fat for energy at the expense of wheat, b) decrease the dietary level of starch and pull it closer to the NP diet, c) decrease dietary starch to CP ratio, and d) provide structural components to the feed, which are all deemed advantageous for wheat-based RP feeding program.

We hypothesized that a reduction in dietary CP in a wheat-based diet will compromise the growth performance of broilers and this will be partially or fully attenuated by adding a moderate amount of insoluble fibre source in the diet, at least some if not all of the selected fibres. This study aimed to investigate the effects of using sugarcane bagasse, lignocellulose-based fibre, soy hulls, or oat hulls as an insoluble fibre source on growth performance, nutrient digestibility, microbiota composition and expression of genes related to digestive enzymes, tight junction proteins, and nutrient transporters in broilers fed RP diets.

## 2 Materials and methods

### 2.1 Animal ethics statement

This experiment was approved by the Animal Ethics Committee of the University of New England (approval No. AEC20–018). All broiler management procedures including health care, husbandry, and use of animals fulfilled the requirements of the Australian Code for the Care and Use of Animals for Scientific Purposes (NHMRC, 2013).

### 2.2 Experimental design and bird management

A total of 672 one-d-old Ross 308 male parental-line broiler chicks were sourced from Aviagen hatchery in Goulburn (NSW, Australia). On d 0, chicks were randomly assigned to 48 floor pens of equal size in an environment-controlled poultry research facility at the University of New England (Armidale, NSW, Australia). Each pen was equipped with a feeder and two nipple drinkers. The birds had ad libitum access to feed and water. The pens were spread with hard wood shavings up to a depth of approximately 7 cm. The lighting and temperature followed the Ross 308 breed guidelines (Aviagen, 2014). On d 10, all birds were weighed and re-assigned to pens of approximately equal weight within 3% of the mean for body weight. The birds were assigned to 6 treatments with 8 replicates of 14 birds per pen. The treatments were: a NP diet, a RP diet (CP reduced by 20 g/kg), and RP diets formulated with either sugarcane bagasse at 20 g/kg, lignocellulose-based product at 10 g/kg, oat hulls at 30 g/kg, or soy hulls at 30 g/kg. The fibres were formulated in the diets but nutrient contributions from the fibres were not included in diet formulations. The basal diet of fibre treatments was the same and the formulations were adjusted by adding Celite, an indigestible component as a filler.

### 2.3 Insoluble fibre

Sugarcane bagasse, a lignocellulose-based product (Arbocell, JRS Pharma GmbH & Co., Rosenberg, Germany), oat hulls, and soy hulls were used as insoluble fibre sources. Sugarcane bagasse was sourced from the FCR consulting group (Brisbane, QLD, Australia). Oat hulls, soy hulls, and lignocellulose-based products were sourced from Ridley Agriproducts Pty Ltd., Pakenham, Victoria, Australia. The fibres were analysed in duplicates for nitrogen by the Dumas method using a LECO FP-2000 automatic nitrogen analyzer (Leco Corp., St. Joseph, MI, USA), dry matter by oven-drying using the method 934.01 of AOAC (1990). GE was determined on a 0.5-g sample using an adiabatic bomb calorimeter (model C7000, IKA-Werke GmbH & Co. KG, Staufen, Germany) with benzoic acid as standard. Soluble and insoluble NSP and their constituent sugars were determined following the procedures previously described (Englyst et al., 1994) with some modifications (Morgan et al., 2019; Theander et al., 1995) using Agilent 8890 GC equipped with Agilent 7693A autosampler (Agilent Technologies Inc., Palo Alto, CA, USA). The lignin content of the fibres was determined by the Klason lignin method which is based on acid hydrolysis of the water-insoluble fraction (Theander and Westerlund, 1986). Starch was determined using the Megazyme Total Starch assay kit (#170203a, Megazyme Int., Ireland Ltd., Wicklow, Ireland).

### 2.4 Diet

The diets were thoroughly mixed and cold-pelleted at 65 °C at the University of New England. The diets contained wheat, sorghum, and soybean meal as major ingredients and were analysed for CP, AA, and apparent metabolizable energy (AME) by near-infrared spectroscopy (NIRS) before diet formulation.

The ingredients and calculated and analysed nutrient composition of the diets are presented in Table 1, Table 2, Table 3, respectively. A common starter crumble (12.55 MJ/kg AME, 23.9% CP) was offered for the first 10 d post–hatch. The treatment grower and finisher diets were offered as 3 mm pellets during d 10 to 24 and d 24 to 35, respectively. The diets were formulated to meet the Ross 308 nutrient specifications (Aviagen, 2019). The CP contributions from supplemental AA were included during diet formulations. The NP diet was balanced for digestible AA by adding supplemental L-Lys, DL-Met, and L-Thr. The RP diet contained 20 g/kg lower CP than the NP diet. The digestible AA in the RP diet was balanced by adding L-Val, L-Arg, L-Ileisoleucine, and L-Gly in addition to L-Lys, DL-Met, and L-Thr. Diets were supplemented with xylanase (Econase XT 25, AB Vista Feed Ingredient, Marlborough, UK) at 1000 BXU/kg and phytase (Quantum Blue, AB Vista Feed Ingredients, Marlborough, UK) at 500 FTU/kg. Nutrient matrix values of phytase were included in diet formulations as recommended by the manufacturer. Vitamin and mineral premixes were added to meet the requirements following manufacturer recommendations from Rabar Pty Ltd (UNE VM, Beaudesert, QLD, Australia). Titanium dioxide was added as an indigestible marker at 0.5% in the grower diets.

**Table 1:** Ingredient composition of the experimental diets.

Ingredients, %	Starter diet	Grower diets			Finisher diets		
		Normal CP^1^	Reduced CP^2^	Reduced CP + Fibre	Normal CP	Reduced CP	Reduced CP + Fibre
Wheat (10.2% CP)	49.110	37.695	46.465	40.135	43.755	52.295	45.925
Sorghum (10.5% CP)	10.000	25.000	25.000	25.000	25.000	25.000	25.000
Soybean meal (47.0% CP)	32.600	30.600	21.800	23.400	25.300	16.600	18.200
Meat meal (52.0% CP)	3.000						
Canola oil	2.600	3.100	1.900	3.700	3.200	2.100	3.900
Insoluble fibre + Celite^3^				3.000			3.000
Limestone	0.940	1.120	1.140	1.120	1.040	1.060	1.050
Dicalcium phosphate	0.410	0.790	0.840	0.860	0.600	0.650	0.670
Sodium chloride	0.220	0.220	0.150	0.150	0.220	0.040	0.050
Sodium bicarbonate	0.100	0.100	0.200	0.200	0.100	0.350	0.350
Choline chloride	0.030	0.040	0.070	0.070	0.030	0.060	0.070
Titanium dioxide		0.500	0.500	0.500			
L-Lys HCl	0.290	0.260	0.510	0.480	0.240	0.490	0.460
DL-Met	0.330	0.280	0.350	0.350	0.250	0.310	0.320
L-Thr	0.150	0.100	0.210	0.200	0.070	0.190	0.180
L-Val	0.010		0.120	0.120		0.110	0.110
L-Arg			0.240	0.220		0.240	0.220
L-Ile			0.100	0.090		0.100	0.090
L-Gly			0.210	0.210		0.210	0.210
Xylanase^4^	0.005	0.005	0.005	0.005	0.005	0.005	0.005
Phytase^5^	0.010	0.010	0.010	0.010	0.010	0.010	0.010
Vitamin premix^6^	0.085	0.080	0.080	0.080	0.080	0.080	0.080
Mineral premix^7^	0.110	0.100	0.100	0.100	0.100	0.100	0.100
Total	100.000						

CP = crude protein.

1 Normal protein diet.

2 Reduced protein diet.

3 Insoluble fibre sources were included in the diets as follows: sugarcane bagasse 2%, lignocellulose 1%, oat hulls 3%, and soy hulls 3%. Celite was added in sugarcane bagasse and lignocellulose treatments at 1% and 2% respectively.

4 Econsase XT, 25 (AB, Vista Feed Ingredient, Marlborough, UK).

5 Quantam Blue 5G (AB, Vista Feed Ingredient, Marlborough, UK).

6 Vitamin premix per kg diet: vitamin A, 12 MIU; vitamin D, 5 MIU; vitamin E, 75 mg; itamin K, 3 mg; nicotinic acid, 55 mg; pantothenic acid, 13 mg; folic acid, 2 mg; riboflavin, 8 mg; cyanocobalamin, 0.016 mg; biotin, 0.25 mg; pyridoxine, 5 mg; thiamine, 3 mg; antioxidant, 50 mg.

7 Mineral premix per kg diet: Cu, 16 mg as copper sulfate; Mn, 60 mg as manganese sulfate; Mn, 60 mg as manganous oxide; I, 0.125 mg as potassium iodide; Se, 0.3 mg; Fe, 40 mg, as iron sulfate; Zn, 50 mg as zinc oxide; Zn, 50 mg as zinc sulfate.

**Table 2:** Calculated nutrient composition of the experimental diets (as-fed basis).

Nutrient compositions, %	Starter diet	Grower diets			Finisher diets		
		Normal CP^1^	Reduced CP^2^	Reduced CP + Fibre^3^	Normal CP	Reduced CP	Reduced CP + Fibre
AME, MJ/kg	12.55	12.87	12.87	12.87	13.18	13.18	13.18
CP	23.9	21.7	19.7	19.7	19.8	17.8	17.8
Crude fat	4.9	5.3	4.2	5.8	5.5	4.4	6.0
Dig Lys^4^	1.28	1.15	1.15	1.15	1.02	1.02	1.02
Dig Met	0.62	0.55	0.58	0.58	0.50	0.53	0.53
Dig Met + Cys	0.95	0.87	0.87	0.87	0.80	0.80	0.80
Dig Thr	0.86	0.77	0.77	0.77	0.68	0.68	0.68
Dig Val	0.96	0.89	0.87	0.87	0.81	0.78	0.78
Dig Arg	1.37	1.23	1.23	1.23	1.09	1.09	1.09
Dig Ile	0.86	0.81	0.78	0.78	0.74	0.70	0.70
Dig Trp	0.28	0.27	0.23	0.23	0.25	0.21	0.21
Dig Leu	1.51	1.50	1.28	1.28	1.37	1.15	1.15
Dig Gly_eq_^5^	1.59	1.34	1.34	1.34	1.22	1.22	1.22
Calcium	0.96	0.86	0.86	0.86	0.78	0.78	0.78
Available P	0.48	0.43	0.43	0.43	0.39	0.39	0.39
Sodium	0.20	0.18	0.18	0.18	0.18	0.18	0.18
Chloride	0.26	0.24	0.25	0.25	0.24	0.18	0.18

CP = crude protein; AME = apparent metabolizable energy.

1 Normal protein diet.

2 Reduced protein diet.

3 Insoluble fibre sources were included in the diets as follows: sugarcane bagasse 2%, lignocellulose 1%, oat hulls 3%, and soy hulls 3%. Celite was added in sugarcane bagasse and lignocellulose treatments at 1% and 2% respectively.

4 Digestible coefficients of AA, for raw materials were determined using AMINODat, 5.0 (Evonik Animal Nutrition).

5 Digestible Gly_eq_ was calculated as follows: Dig Gly_eq_ = Dig Gly + (Dig Ser × 0.7143).

**Table 3:** Analysed nutrient composition of the experimental grower and finisher diets (as-fed basis).

Nutrient Compositions, %	Grower diets						Finisher diets					
	Normal	Reduced	Reduced	Reduced	Reduced	Reduced	Normal	Reduced	Reduced	Reduced	Reduced	Reduced
	CP^1^	CP^2^	CP + SB	CP + LC	CP + OH	CP + SH	CP	CP	CP + SB	CP + LC	CP + OH	CP + SH
DM	87.6	87.5	87.9	87.8	87.9	87.8	87.1	87.2	87.8	88.2	87.8	87.5
GE^3^, MJ/kg	16.76	16.44	16.66	16.45	16.85	16.75	16.64	16.43	16.69	16.58	16.77	16.78
CP	21.3	19.3	19.4	19.0	19.4	19.7	19.4	17.5	17.5	17.0	17.3	17.4
Starch	35.8	41.8	36.5	37.9	36.2	37.8	40.6	46.7	42.2	41.3	39.9	41.1
Starch:CP	1.68	2.17	1.88	1.99	1.87	1.92	2.09	2.67	2.41	2.43	2.31	2.36
Lys	1.23	1.17	1.20	1.18	1.06	1.26	1.05	1.02	1.05	1.05	1.05	1.03
Met	0.44	0.50	0.53	0.45	0.45	0.49	0.42	0.47	0.42	0.43	0.48	0.43
Thr	0.85	0.81	0.82	0.83	0.77	0.84	0.74	0.70	0.72	0.72	0.72	0.72
Val	1.03	0.96	0.96	0.99	0.92	1.00	0.91	0.85	0.88	0.88	0.87	0.88
Arg	1.27	1.20	1.23	1.24	1.13	1.24	1.09	1.09	1.08	1.09	1.10	1.08
Ile	0.96	0.86	0.86	0.89	0.81	0.89	0.83	0.75	0.78	0.78	0.78	0.78
Leu	1.71	1.44	1.44	1.50	1.39	1.51	1.54	1.31	1.34	1.34	1.33	1.34
Gly	0.87	0.90	0.93	0.93	0.87	1.04	0.77	0.82	0.85	0.87	0.86	0.86
Ser	1.02	0.83	0.84	0.88	0.80	0.88	0.90	0.76	0.77	0.77	0.77	0.78
Phe	1.07	0.88	0.87	0.92	0.84	0.92	0.94	0.79	0.81	0.81	0.80	0.81
His	0.55	0.46	0.46	0.48	0.44	0.48	0.49	0.41	0.42	0.42	0.42	0.42
Tyr	0.54	0.43	0.46	0.45	0.40	0.45	0.46	0.40	0.38	0.36	0.40	0.39
Pro	1.32	1.18	1.14	1.19	1.12	1.19	1.23	1.14	1.12	1.12	1.12	1.11
Ala	1.00	0.85	0.85	0.88	0.82	0.90	0.91	0.78	0.79	0.78	0.78	0.79
Asp	1.95	1.48	1.52	1.60	1.42	1.62	1.64	1.31	1.34	1.32	1.33	1.35
Glu	4.33	3.73	3.65	3.84	3.55	3.80	3.93	3.59	3.54	3.48	3.52	3.51

CP = crude protein; SB = 2% sugarcane bagasse and 1% celite powder; LC = 1% lignocellulose and 2% celite powder; OH = 3% oat hulls; SH = 3% soy hulls; DM = dry matter; GE = gross energy.

1 Normal protein diet.

2 Reduced protein diet.

3 GE, calculated using an adiabatic bomb calorimeter (model C7000, IKA-Werke GmbH & Co. KG, Staufen, Germany).

### 2.5 Growth performance

Birds and feed were weighed on arrival and on d 10, 24, and 35, and mortality was recorded daily. The FI, WG, and mortality adjusted FCR were determined during the experimental periods.

### 2.6 Water intake

**Fig. 1:**
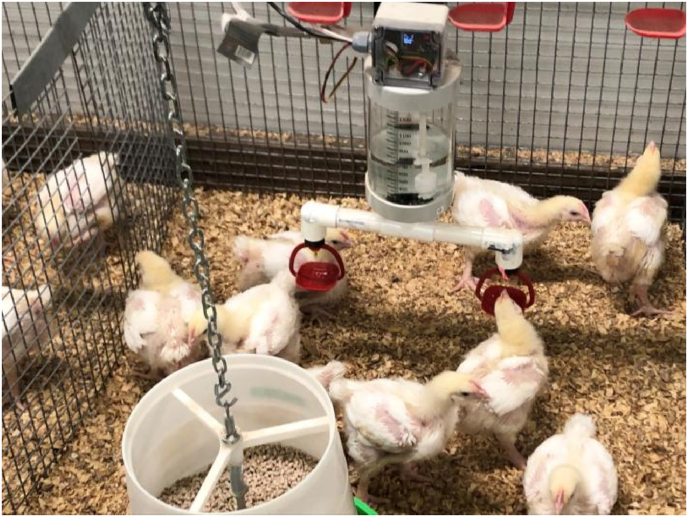
An automated drinker system for measuring water intake.

An automated drinker system was developed (Fig. 1) to measure water intake (WI) at two time points (d 10–24 and 24–35). The system consisted of a 1.4 L reservoir with a microcontroller to monitor water consumption. Sensors in the reservoir triggered the opening of a solenoid valve to allow it to refill when it was below a minimum level. A flow meter on the inlet line measured the amount of water taken to refill the reservoir. This data, along with a cumulative count of the water use since the beginning of the trial and the number of refills was transmitted wirelessly to a computer. Information in the database was collected and used to calculate WI in each pen.

### 2.7 Carcass traits and internal organs

On d 24 and 35, three birds were sampled per pen to measure the relative weights of gizzard, gizzard contents, and gizzard pH. On d 35, the sampled birds were also used for measuring the relative weights of breast, thigh, drumstick, abdominal fat, and pancreas. The relative weights of carcass cuts and internal organs were calculated as mass per unit (g/kg) of live body weight. The pH of gizzard content was measured in situ by inserting a pH probe (Hanna FC200, Hanna Instruments, Woonsocket, RI, USA), connected to a pH meter (OHAUS ST-300G, Ohaus, Parsippany, NJ, USA), directly into the digesta ensuring the probe did not touch the intestinal wall. The procedure was repeated three times and an average pH value of the three readings was taken.

### 2.8 Nutrient digestibility

On d 24, three birds were randomly sampled per pen, individually weighed, and euthanised by electrical stunning (MEFE CAT 44N, Mitchell Engineering Food Equipment, Clontarf, QLD, Australia) and cervical dislocation. The carcass was opened to collect the contents from the distal jejunum and distal ileum by gently squeezing the contents into 50 mL plastic containers. The contents were pooled from each section and stored at *-*20 °C until further processing to measure the apparent digestibility of CP, starch, and AA. The frozen samples were freeze-dried (Alpha 1–4 LDplus, Marin Christ, Osterode am Harz, Lower Saxony, Germany) and ground to pass through a 0.5-mm screen. Grounded feed and intestinal contents were analysed in duplicates for nitrogen by the Dumas method (method 968.06) of AOAC (1990) using a LECO FP-2000 automatic nitrogen analyzer (Leco Corp., St. Joseph, MI, USA). The nitrogen value was multiplied by 6.25 to calculate the CP level in the feed and intestinal content. AA profiles in feed and intestinal contents were measured by 24-h liquid hydrolysis at 110 °C in 6 mol/L HCl and then AA were analyzed by ultra-performance liquid chromatography (Waters Corp., Milford, MA, USA) (Boogers et al., 2008). The starch contents of feed and digesta samples were measured using the Megazyme Total Starch assay kit. The GE of ground feed and intestinal contents were determined in duplicates on a 0.5-g sample using an adiabatic bomb calorimeter with benzoic acid as standard. Titanium dioxide (TiO_2_) was measured in duplicate for diets and digesta samples by colorimetric method (Short et al., 1996). The ground feed and freeze-dried samples were oven-dried using the method 934.01 of AOAC (1990).

The apparent digestibility coefficient of nutrients was calculated by using the following equation:

Xdigestibilitycoefficient=100-(Tidiet/Tidigesta×Xdigesta/Xdiet),

where *X* refers to the nutrient under analysis.

### 2.9 DNA extraction and quantification of bacteria

On d 24, contents of the caeca and ileum from three birds per pen were collected and pooled in 50-mL containers for each section separately. Then, sub-samples of the pooled digesta were aliquoted into 2-mL Eppendorf tubes and snap-frozen in liquid N_2_. The samples were stored at −20 °C until analysis. The DNA of cecal and ileal contents were extracted using PowerFecal QIAcube HT kit (Qiagen Inc., Hilden, Germany), and relative amounts of *Bacillus* spp.*, Bacteroides* spp.*, Bifidobacterium* spp.*,* Enterobacteriaceae*, Lactobacillus* spp.*, Ruminococcus* spp., and total bacteria, expressed as log_10_ (genomic DNA copies) per g of cecal contents were quantified following the procedures described by Kheravii et al. (2017). The quantitative real-time PCR (Rotor Gene-6000, Corbett, Sydney, Australia) was employed to determine the bacterial population. The specific 16S rRNA primers were used for the quantification of different bacterial populations and are presented in Table S1.

### 2.10 Sample collection and processing for gene expression studies

On d 24, jejunal and pancreatic tissues were collected from two birds per pen to measure gene expression of digestive enzymes, tight junction proteins, and nutrient transporters. Around 2 cm of tissues from the mid pancreas and proximal jejunum were excised, carefully flushed with chilled sterile phosphate-buffered saline (PBS), and dipped in 2-mL Eppendorf tubes containing RNA later (Qiagen, Hilden, Germany). The tissues were stored at 4 °C for the first 4 to 6 h and then at −20 °C until processed for RNA extraction. For total RNA extraction, tissues were homogenized for 3 to 5 min using a Tissuelyzer II following the manufacturer's instructions. The total RNA collected was purified using the ISOLATE II RNA Mini kit (Bioline [Aust] Pty Ltd., Sydney, Australia) with the inclusion of a DNase I digestion step to remove any remaining genomic DNA following the manufacturer's instructions. Total RNA quantity and purity were determined using a NanoDrop ND-8000 spectrophotometer (Thermo Fisher Scientific Inc., Waltham, MA, USA). RNA integrity was measured by Agilent 2100 Bioanalyzer (Agilent Technologies Inc., Waldbronn, Germany) using an RNA 6000 Nano kit. The RNA samples were considered of high integrity if the RIN was higher than 7.0 for jejunal and 6.0 for pancreatic tissues. The extracted RNA of the samples was reverse transcribed to cDNA using the SensiFAST cDNA Synthesis kit (Bioline [Aust] Pty Ltd., Sydney, Australia) in a Rotor Gene-6000 real-time PCR instrument as per the manufacturer's instructions. The cDNA was diluted ten times with nuclease-free water and stored at −20 °C until further analysis. In addition, around 2 cm of tissue from the mid-portion of the proventriculus was collected and processed in the same way as the other tissues to investigate the expression of two additional digestive enzyme genes (*Pep A* and *Pep C*). The genes determined in the pancreas, proventriculus, and jejunum are summarised in Table S1.

### 2.11 Real-time quantitative PCR (RT-qPCR)

Real-time quantitative PCR was performed using a SYBR Green kit SensiFAST SYBR No-ROX (Bioline [Aust] Pty Ltd., Sydney, Australia) on a Rotor Gene-6000 real-time PCR machine. The PCR reaction was carried out in a volume of 10 μL containing 5 μL of 2 × SensiFAST, 400 nmol/L of each primer, 2.2 μL of nuclease-free water, and 2 μL of 10 × diluted cDNA sample. To determine two suitable reference genes for the analysis, the geNorm module in qBase + software version 3.0 (Biogazelle, Zwijnbeke, Belgium) was used to calculate the gene expression stability (geNorm M) from ten widely used house-keeping genes. Based on the expression stability of the reference genes, *SDHA* and *GAPDH* were chosen to normalise the target genes in the pancreas as they had the lowest M values compared to the other reference genes (M = 0.263). Similarly, *ACTB* and *GAPDH* were chosen for jejunum (M = 0.180) and *HMBS* and *YWHAZ* were chosen for proventriculus (M = 0.270) as housekeeping genes to normalize the target genes.

The relative quantification of genes using the arithmetic mean method in the qBase + software was exported to the SAS 9.3 package (SAS Institute Inc., Cary, NC, USA) for further analysis. Primers for qPCR were either sourced from published papers or designed using the NCBI Primer-BLAST Tool (https://www.ncbi.nlm.nih.gov/tools/primer-blast/) as presented in Table S3. All the primers used in this study were checked for specificity using an Agilent DNA 1000 kit (Agilent Technologies Inc., Waldron, Germany) on an Agilent 2100 Bioanalyzer (Agilent Technologies Inc., Waldron, Germany).

### 2.12 Statistical analysis

The data were analysed by one-way ANOVA using JMP statistical software version 14 (SAS Institute Inc., Cary, NC, USA). Pen served as the experimental unit. The model used was as follows:

$$Y_{ij}=\mu +T_{i}+\varepsilon_{ij}\text{,}$$

where *Y*_*ij*_ is the value for each random variable, *μ* is the overall mean, *T*_*i*_ is the fixed effect corresponding to the *i-*th treatment such that Σ*T*_*i*_ = 0, and *ε*_*ij*_ is the random error. Significance was determined at *P* < 0.05 using Tukey's HSD and a tendency was declared at 0.05 < *P* < 0.10.

## 3 Results

The analysed CP content in the diets was close to the calculated values (Table 2, Table 3). The difference in CP levels between the NP and RP diets was similar in the analysed and calculated compositions.

### 3.1 Composition of insoluble fibres

The composition of different insoluble fibres has been shown in Table 4. Lignocellulose contained the highest concentration of crude fibre (65.7%) followed by bagasse (45.2%), soy hulls (37.7%), and oat hulls (31.0%). Bagasse contained the highest concentration of INSP (53.6%) followed by lignocellulose (48.9%), oat hulls (47.4%), and soy hulls (45.1%). Lignocellulose contained the highest concentration of lignin (26.8%) followed by bagasse (18.1%), oat hulls (15.2%), and soy hulls (3.8%). Oat hulls contained 6.2% starch whereas no starch was detected on the other fibre sources. Soy hulls contained the highest concentration of CP (9.3%) followed by oat hulls (3.1%), bagasse (1.0%), and lignocellulose (0.3%).

**Table 4:** Composition of sugarcane bagasse, lignocellulose, oat hulls, and soy hulls (%).

Insoluble fibre		Rhamnose	Fucose	Ribose	Arabinose	Xylose	Mannose	Galactose	Glucose	Total	Lignin	DM	GE^1^ MJ/kg	CP	CF	Starch	Ash
Sugarcane bagasse	Free sugars	ND	ND	0.009	0.069	0.431	0.041	0.118	0.210	0.879	18.1	92.7	17.14	1.0	45.2	ND	2.5
	SNSP	0.005	ND	0.002	0.022	0.025	0.035	0.050	0.085	0.201							
	INSP	ND	0.049	0.158	2.336	19.020	0.145	1.080	37.250	53.570							
	Total NSP	ND	0.049	0.160	2.358	19.040	0.180	1.130	37.340	53.780							
Lignocellulose^2^	Free sugars	ND	ND	0.007	0.021	0.050	0.072	0.100	0.058	0.305	26.8	93.2	17.64	0.3	65.7	ND	0.5
	SNSP	0.007	ND	0.002	0.030	0.012	0.149	0.238	0.041	0.430							
	INSP	0.069	0.054	0.160	1.043	4.496	7.795	2.781	38.170	48.940							
	Total NSP	0.076	0.054	0.162	1.073	4.508	7.944	3.019	38.210	49.370							
Oat hulls	Free sugars	0.010	ND	0.011	0.034	0.051	0.171	0.092	0.797	1.166	15.2	93.5	16.39	3.1	31.0	6.2	5.1
	SNSP	0.005	ND	0.003	0.066	0.104	0.037	0.070	0.210	0.441							
	INSP	ND	0.212	0.128	3.470	24.380	0.050	1.344	23.75	47.430							
	Total NSP	ND	0.212	0.131	3.536	24.490	0.087	1.414	23.96	47.870							
Soy hulls	Free sugars	0.016	ND	0.011	0.069	0.027	0.132	0.430	0.638	1.323	3.8	91.1	15.82	9.3	37.7	ND	4.5
	SNSP	0.065	0.013	0.009	0.717	0.044	1.380	0.856	0.037	2.793							
	INSP	0.321	0.330	0.162	3.462	6.129	2.989	2.414	34.490	45.060							
	Total NSP	0.386	0.343	0.171	4.179	6.173	4.369	3.270	34.520	47.860							

DM = dry matter; GE = gross energy; CP = crude protein; CF = crude fibre; SNSP = soluble non-starch polysaccharides; INSP = insoluble non-starch polysaccharides; ND = not detected.

1 GE, calculated using an adiabatic bomb calorimeter (model C7000, IKA-Werke GmbH & Co. KG, Staufen, Germany).

2 Arbocell from JRS, Pharma GmbH & Co. (Rosenberg, Germany).

### 3.2 Growth performance

The performance of birds exceeded the Ross 308 performance objectives (Aviagen, 2019) for FI, WG, and FCR (Table 5). During d 10 to 35, the average FI, WG, and FCR were 3218 g, 2220 g, and 1.450 respectively. Mortality was low (<2.5%) and not affected by the dietary treatments (data not reported). The effects of RP diets with insoluble fibres on the growth performance of broilers are presented in Table 5. Dietary treatments tended (*P* = 0.067) to affect FI during d 10 to 24 and the highest FI during the period was observed in the birds offered a NP diet. Based on the overall ANOVA, dietary treatments had a significant effect on FI (*P* < 0.001) during d 24 to 35 and 10 to 35. During these periods, paired comparison results showed that the reduction in dietary CP decreased (*P* < 0.05) the FI of birds. The birds offered a RP diet with either bagasse, lignocellulose, or oat hulls had no effect (*P* = 0.067) on FI compared to those offered a RP diet without fibres. The birds offered a RP diet with soy hulls had higher (*P* < 0.05) FI compared to those offered a RP diet during d 24 to 35 and similar FI as NP treatment in both d 24 to 35 and 10 to 35.

**Table 5:** Growth performance of broilers offered reduced protein diets with insoluble fibres.

Treatments	FI, g/bird			WG, g/bird			FCR, g/g		
	d 10–24	d 24–35	d 10–35	d 10–24	d 24–35	d 10–35	d 10–24	d 24–35	d 10–35
Normal CP^1^	1412	1895^a^	3307^a^	1084^a^	1214^a^	2298^a^	1.302^b^	1.561	1.439^d^
Reduced CP^2^	1380	1817^b^	3198^bc^	1041^bc^	1150^c^	2190^cd^	1.326^a^	1.581	1.460^a^
Reduced CP + SB	1368	1846^ab^	3214^bc^	1053^bc^	1174^bc^	2227^bc^	1.300^b^	1.573	1.443^cd^
Reduced CP + LC	1370	1787^b^	3158^c^	1031^c^	1146^c^	2176^cd^	1.330^a^	1.560	1.451^abc^
Reduced CP + OH	1359	1792^b^	3151^c^	1026^c^	1139^c^	2166^d^	1.324^a^	1.573	1.455^ab^
Reduced CP + SH	1389	1890^a^	3278^ab^	1069^ab^	1194^ab^	2263^ab^	1.299^b^	1.583	1.448^bcd^
SEM	12.6	19.0	27.9	9.8	12.0	18.3	0.0053	0.0062	0.0041
*P-*value	0.067	<0.001	<0.001	<0.001	<0.001	<0.001	<0.001	0.072	<0.001

FI = feed intake; WG = weight gain; FCR = feed conversion ratio; CP = crude protein; SB = 2% sugarcane bagasse and 1% celite powder; LC = 1% lignocellulose and 2% celite powder; OH = 3% oat hulls; SH = 3% soy hulls; SEM = standard error of the mean.

Within each treatment factor, means in the same column with a different superscript differ significantly (*P* < 0.05), *n* = 8.

1 Normal protein diet.

2 Reduced protein diet.

Based on the ANOVA result, dietary treatments had a significant effect on WG (*P* < 0.001) in all the growing periods. The reduction in dietary CP decreased (*P* < 0.001) WG of birds in all the growth periods. The birds offered a RP diet with soy hulls had a higher (*P* < 0.001) WG compared to those offered a RP diet and similar WG to those offered RP + bagasse and NP diets during d 10 to 24, 24 to 35, and 10 to 35. Dietary treatments led to a significant difference in FCR (*P* < 0.001) during d 10 to 24 and 10 to 35 based on the overall ANOVA. During these periods, the reduction in dietary CP increased (*P* < 0.05) the FCR of birds except for 24 to 35 d which tended to increase (*P* = 0.072) FCR. The birds offered a RP diet with soy hulls or bagasse had a better FCR (*P* < 0.001) compared to those offered a RP diet without fibre and similar FCR to those offered a NP diet.

### 3.3 Carcass cuts and internal organs

The effects of RP diets with insoluble fibres on the relative organ weights of broilers are presented in Table 6. ANOVA showed that dietary treatments had a significant effect (*P* = 0.048) on relative abdominal fat weight. The reduction in dietary CP increased (*P* < 0.05) the relative abdominal fat weight of birds. The birds offered a RP diet with insoluble fibres (bagasse, lignocellulose, oat hulls, or soy hulls) had no effect (*P* > 0.05) on relative abdominal fat weight compared to those offered a RP diet. Dietary treatments tended (*P* = 0.058) to affect relative pancreas weight and the highest weight was observed in the NP treatment. There was no effect of dietary treatments on the relative weights of breast (*P* = 0.744), thigh (*P* = 0.710), and drumstick (*P* = 0.153).

**Table 6:** Relative organ weights of broilers offered reduced protein diets with insoluble fibres on d 35 (g/kg).

Treatments	Breast	Thigh	Drumstick	Abdominal fat pad	Pancreas
Normal CP^1^	162.7	99.96	90.45	11.68^b^	1.70
Reduced CP^2^	159.4	99.54	91.09	13.67^a^	1.56
Reduced CP + SB	158.4	99.80	92.43	13.65^a^	1.62
Reduced CP + LC	158.9	99.90	91.34	13.67^a^	1.50
Reduced CP + OH	158.3	102.60	93.51	13.17^ab^	1.69
Reduced CP + SH	158.6	99.90	90.54	12.88^ab^	1.67
SEM	2.29	1.503	0.912	0.501	0.054
*P-*value	0.744	0.710	0.153	0.048	0.058

CP = crude protein; SB = 2% sugarcane bagasse and 1% celite powder; LC = 1% lignocellulose and 2% celite powder; OH = 3% oat hulls; SH = 3% soy hulls; SEM = standard error of the mean.

Within each treatment factor, means in the same column with a different superscript differ significantly (*P* < 0.05), *n* = 8.

1 Normal protein diet.

2 Reduced protein diet.

ANOVA showed that dietary treatments led to significant effects in relative gizzard weight (*P* = 0.039) and gizzard contents (*P* = 0.019) on d 24 and 35 (*P* < 0.001, for both the parameters) as shown in Table 7. The reduction in dietary CP had no effect (*P* > 0.05) on the relative weight of gizzard at both time points. On d 24, the birds offered a RP diet with bagasse or oat hulls had a higher (*P* < 0.05) relative weight of gizzard compared to those offered a NP diet. On d 35, the birds offered a RP diet with oat hulls or bagasse had a higher (*P* < 0.05) relative weight of gizzard compared to those offered a RP diet. The addition of bagasse in RP diets increased (*P* < 0.05) the relative contents of gizzard on d 35. There was no effect (*P* > 0.05) of dietary treatments on gizzard pH on d 24 (*P* = 0.272) and 35 (*P* = 0.267) of ages.

**Table 7:** Relative gizzard weight, gizzard contents and gizzard pH of broilers offered reduced protein diets with insoluble fibres.

Treatments	d 24			d 35		
	Gizzard weight, g/kg	Gizzard contents, g/kg	Gizzard pH	Gizzard weight, g/kg	Gizzard contents, g/kg	Gizzard pH
Normal CP^1^	17.82^b^	10.39^a^	3.03	11.49^bc^	4.93^bc^	3.57
Reduced CP^2^	18.25^ab^	8.70^b^	3.03	11.37^c^	4.96^bc^	3.60
Reduced CP + SB	19.35^a^	9.71^ab^	2.97	12.36^ab^	6.72^a^	3.58
Reduced CP + LC	18.10^ab^	8.71^b^	2.99	10.78^c^	4.22^c^	3.61
Reduced CP + OH	19.50^a^	8.92^ab^	3.02	13.34^a^	6.33^ab^	3.32
Reduced CP + SH	17.82^b^	9.26^ab^	3.04	11.40^bc^	5.16^bc^	3.42
SEM	0.503	0.406	0.085	0.344	0.493	0.103
*P-*value	0.039	0.019	0.272	<0.001	<0.001	0.267

CP = crude protein; SB = 2% sugarcane bagasse and 1% celite powder; LC = 1% lignocellulose and 2% celite powder; OH = 3% oat hulls; SH = 3% soy hulls; SEM = standard error of the mean.

Within each treatment factor, means in the same column with a different superscript differ significantly (*P* < 0.05), *n* = 8.

1 Normal protein diet.

2 Reduced protein diet.

### 3.4 Water intake

The effects of RP diets with insoluble fibres on WI, and water-to-feed intake ratio (WI:FI) of broilers are presented in Table 8. ANOVA showed that dietary treatments had significant effects on WI (*P* < 0.001, for all the growing periods) and WI:FI during d 10 to 24 and 10 to 35 (*P* < 0.001). The reduction in dietary CP decreased (*P* < 0.05) WI and WI:FI in all the phases. The birds offered a RP diet with insoluble fibres (bagasse, lignocellulose, oat hulls, or soy hulls) had no effect (*P* > 0.05) on WI and WI:FI compared to those offered a RP diet.

**Table 8:** Water intake and water-to-feed intake ratio of broilers offered reduced protein diets with insoluble fibres.

Treatments	Water intake, g/bird			Water-to-feed intake ratio^3^		
	d 10–24	d 24–35	d 10–35	d 10–24	d 24–35	d 10–35
Normal CP^1^	3108^a^	3650^a^	6757^a^	2.19^a^	1.93^a^	2.05^a^
Reduced CP^2^	2767^bc^	3216^bc^	5983^bc^	2.00^b^	1.78^b^	1.88^b^
Reduced CP + SB	2800^bc^	3304^bc^	6105^bc^	2.04^b^	1.79^b^	1.89^b^
Reduced CP + LC	2768^bc^	3175c	5943c	2.03^b^	1.78^b^	1.89^b^
Reduced CP + OH	2726^c^	3290^bc^	6016^bc^	2.01^b^	1.84^b^	1.91^b^
Reduced CP + SH	2875^b^	3419^ab^	6294^b^	2.07^b^	1.81^b^	1.92^b^
SEM	33.7	56.3	79.5	0.026	0.027	0.023
*P-*value	<0.001	<0.001	<0.001	<0.001	0.002	<0.001

WI = water intake; WI:FI = water-to-feed intake ratio; CP = crude protein; SB = 2% sugarcane bagasse and 1% celite powder; LC = 1% lignocellulose and 2% celite powder; OH = 3% oat hulls; SH = 3% soy hulls; SEM = standard error of the mean.

Within each treatment factor, means in the same column with a different superscript differ significantly (*P* < 0.05), *n* = 8.

1 Normal protein diet.

2 Reduced protein diet.

3 Feed intake data taken from Table 7 to calculate the ratio.

### 3.5 Nutrient digestibility

The effects of RP diets with insoluble fibres on apparent starch and CP digestibility coefficients in the distal jejunum and distal ileum and GE digestibility coefficient in the distal ileum of broilers are presented in Table 9. ANOVA showed that the dietary treatments had a significant effect (*P* = 0.003) on the starch digestibility coefficient in the distal ileum but not in the distal jejunum (*P* = 0.212). The reduction in dietary CP increased (*P* < 0.05) the starch digestibility coefficient in the distal ileum. The birds offered a RP diet with either bagasse or soy hulls had a lower (*P* < 0.05) apparent ileal digestibility (AID) coefficient of starch in the distal ileum compared to those offered a RP diet without fibre and a similar starch digestibility coefficient to those offered a NP diet.

**Table 9:** Apparent starch, crude protein and gross energy digestibility coefficients in the intestine of broilers offered reduced protein diets with insoluble fibres on d 24.

Treatment	Starch digestibility coefficient		CP digestibility coefficient		GE^1^ digestibility coefficient
	Distal jejunum	Distal ileum	Distal jejunum	Distal ileum	Distal ileum
Normal CP^2^	0.864	0.936^d^	0.590^ab^	0.812	0.734^ab^
Reduced CP^3^	0.891	0.962^a^	0.639^a^	0.814	0.751^a^
Reduced CP + SB	0.872	0.947^bcd^	0.563^b^	0.798	0.729^b^
Reduced CP + LC	0.866	0.952^abc^	0.600^ab^	0.796	0.721^b^
Reduced CP + OH	0.870	0.956^ab^	0.606^ab^	0.807	0.728^b^
Reduced CP + SH	0.869	0.940^cd^	0.608^ab^	0.800	0.729^b^
SEM	0.0082	0.0051	0.0142	0.0071	0.0063
*P-*value	0.212	0.003	0.020	0.358	0.002

CP = crude protein; GE = gross energy; SB = 2% sugarcane bagasse and 1% celite powder; LC = 1% lignocellulose and 2% celite powder; OH = 3% oat hulls; SH = 3% soy hulls; SEM = standard error of the mean.

Within each treatment factor, means in the same column with a different superscript differ significantly (*P* < 0.05), *n* = 8.

1 GE, calculated using an adiabatic bomb calorimeter (model C7000, IKA-Werke GmbH & Co. KG, Staufen, Germany).

2 Normal protein diet.

3 Reduced protein diet.

ANOVA showed that the dietary treatments had a significant effect (*P* = 0.020) on the CP digestibility coefficient in the distal jejunum but not in the distal ileum (*P* = 0.358). The birds offered a RP diet with bagasse had a lower (*P* < 0.05) CP digestibility coefficient in distal jejunum compared to those offered a RP diet.

ANOVA showed that the dietary treatments had a significant effect (*P* = 0.002) on the GE digestibility coefficient in the distal ileum. The birds offered a RP diet with either of the four insoluble fibres had a lower (*P* < 0.05) GE digestibility coefficient compared to those offered a RP diet without fibre but a similar GE digestibility coefficient to those offered a NP diet.

The effect of RP diets with insoluble fibres on AID coefficients of AA on d 24 has been presented in Table 10. ANOVA showed that the dietary treatments affected AID coefficients of Met (*P* = 0.040), Tyr (*P* = 0.022), His (*P* = 0.017), Gly (*P* = 0.018), Ser (*P* = 0.017), and Asp (*P* = 0.001), and tended to affect AID of Pro (*P* = 0.057). The reduction in dietary CP increased (*P* < 0.05) the AID coefficients of Met and Gly, and decreased (*P* < 0.05) the AID coefficient of Asp, but had no effects (*P* > 0.05) on the AID coefficients of other AA. There was no effect (*P* > 0.05) of adding either of the four insoluble fibres to the RP diet on AID of Met, Gly, Tyr, Ser, and Asp except that the birds offered a RP diet with oat hulls had lower (*P* < 0.05) AID of Tyr, Ser, and Asp compared to those offered a RP diet. The birds offered a RP diet with bagasse or oat hulls had lower (*P* < 0.05) AID coefficients of His compared to those offered a RP diet.

**Table 10:** Apparent ileal digestibility coefficients of amino acids as affected by reduced protein diets with insoluble fibres on d 24.

Treatments	Lys	Met	Thr	Val	Iso	Arg	Phe	Tyr	His	Leu	Gly	Ser	Ala	Pro	Asp	Glu
Normal CP^1^	0.878	0.934^b^	0.778	0.808	0.824	0.892	0.837	0.824^a^	0.835^a^	0.806	0.782^c^	0.802^a^	0.779	0.824	0.794^a^	0.871
Reduced CP^2^	0.885	0.945^a^	0.793	0.817	0.828	0.900	0.829	0.808^ab^	0.824^ab^	0.800	0.812^ab^	0.784^ab^	0.770	0.827	0.770^b^	0.870
Reduced CP + SB	0.877	0.945^a^	0.776	0.800	0.813	0.891	0.812	0.804^abc^	0.806^c^	0.782	0.801^abc^	0.766^bc^	0.749	0.802	0.754^bc^	0.854
Reduced CP + LC	0.877	0.942^a^	0.778	0.806	0.818	0.895	0.822	0.800^bc^	0.811^bc^	0.788	0.801^abc^	0.778^bc^	0.753	0.808	0.767^b^	0.860
Reduced CP + OH	0.865	0.937^ab^	0.769	0.798	0.807	0.887	0.813	0.784^c^	0.805^c^	0.786	0.794^bc^	0.761^c^	0.752	0.813	0.745^c^	0.861
Reduced CP + SH	0.882	0.939^ab^	0.787	0.812	0.822	0.895	0.825	0.796^bc^	0.814^bc^	0.793	0.816^a^	0.777^bc^	0.763	0.811	0.774^ab^	0.859
SEM	0.0061	0.0032	0.0083	0.0071	0.0071	0.0052	0.0074	0.0081	0.0062	0.0082	0.0073	0.0081	0.0094	0.0062	0.0071	0.0052
*P-*value	0.265	0.040	0.297	0.428	0.325	0.546	0.119	0.022	0.017	0.322	0.018	0.017	0.220	0.057	0.001	0.225

CP = crude protein; SB = 2% sugarcane bagasse and 1% celite powder; LC = 1% lignocellulose and 2% celite powder; OH = 3% oat hulls; SH = 3% soy hulls; SEM = standard error of the mean.

Within each treatment factor, means in the same column with a different superscript differ significantly (*P* < 0.05), *n* = 8.

1 Normal protein diet.

2 Reduced protein diet.

### 3.6 Microflora

The effect of RP diets with insoluble fibres on bacterial load (log_10_ [genomic DNA copies]/g) in ileal contents of broilers on d 24 are presented in Table 11. Overall ANOVA showed that the dietary treatments had a significant effect (*P* = 0.022) on *Lactobacillus* counts and a tendency was observed for *Bifidobacteria* (*P* = 0.095), *Ruminococcus* (*P* = 0.058), and total bacteria (*P* = 0.056) counts in the ileal contents. The birds offered a RP diet with oat hulls had lower (*P* < 0.05) *Lactobacillus* counts in the ileal contents compared to those offered a RP diet with lignocellulose or soy hulls. The birds offered a RP diet with either oat hulls or bagasse had the lowest counts of total bacteria in the ileal contents compared to others.

**Table 11:** Bacterial load (log_10_ genomic DNA copies/g) in ileal contents of broilers offered reduced protein diets with insoluble fibres on d 24.

Treatments	*Bacillus*	*Bacteroides*	*Bifidobacteria*	*Enterobacteria*	*Lactobacillus*	*Ruminococcus*	Total bacteria
Normal CP^1^	6.84	4.1	7.77	7.92	9.55^ab^	4.63	10.58
Reduced CP^2^	6.88	4.13	7.75	7.45	9.54^ab^	4.83	10.59
Reduced CP + SB	6.80	4.14	7.58	7.04	9.42^ab^	4.91	10.37
Reduced CP + LC	6.87	4.47	7.84	7.47	9.66^a^	4.84	10.61
Reduced CP + OH	6.90	4.13	7.60	7.25	9.31^b^	4.67	10.38
Reduced CP + SH	6.91	4.73	7.88	7.86	9.68^a^	4.43	10.65
SEM	0.079	0.212	0.088	0.376	0.081	0.116	0.078
*P-*value	0.928	0.215	0.095	0.545	0.022	0.058	0.056

CP = crude protein; SB = 2% sugarcane bagasse and 1% celite powder; LC = 1% lignocellulose and 2% celite powder; OH = 3% oat hulls; SH = 3% soy hulls; SEM = standard error of the mean.

Within each treatment factor, means in the same column with a different superscript differ significantly (*Pn* = 8.

1 Normal protein diet.

2 Reduced protein diet.

The effect of RP diet with insoluble fibres on bacterial load in caecal contents of broilers on d 24 are presented in Table 12. Overall ANOVA showed the dietary treatments had a significant effect on *Lactobacillus* (*P* = 0.047) and *Ruminococcus* (*P* = 0.013) counts, and a tendency (*P* = 0.076) was observed for *Bacteroides* counts in the caecal contents. The birds offered a RP diet with either bagasse or oat hulls had lower (*P* < 0.05) *Lactobacillus* counts in the caecal contents compared to those offered a RP diet and a RP diet with soy hulls.

**Table 12:** Bacterial load (log_10_ [genomic DNA copies]/g) in caecal contents of broilers offered reduced protein diets with insoluble fibres on d 24.

Treatments	*Bacillus*	*Bacteroides*	*Bifidobacteria*	*Enterobacteria*	*Lactobacillus*	*Ruminococcus*	Total bacteria
Normal CP^1^	7.51	5.68	9.12	7.96	10.18^ab^	9.52^b^	11.66
Reduced CP^2^	7.72	5.75	9.12	7.99	10.28^a^	9.67^ab^	11.89
Reduced CP + SB^3^	7.85	5.85	9.15	8.00	9.98^b^	9.65^ab^	11.87
Reduced CP + LC^4^	7.88	5.82	9.10	8.07	10.05^ab^	9.73^a^	11.93
Reduced CP + OH^5^	7.64	5.87	9.10	7.57	9.98^b^	9.75^a^	11.88
Reduced CP + SH^6^	8.12	5.80	9.33	8.33	10.28^a^	9.68^ab^	11.90
SEM	0.197	0.048	0.108	0.256	0.088	0.045	0.070
*P-*value	0.348	0.076	0.664	0.478	0.047	0.013	0.113

CP = crude protein; SB = 2% sugarcane bagasse and 1% celite powder; LC = 1% lignocellulose and 2% celite powder; OH = 3% oat hulls; SH = 3% soy hulls; SEM = standard error of the mean.

Within each treatment factor, means in the same column with a different superscript differ significantly (*P* < 0.05), *n* = 8.

1 Normal protein diet.

2 Reduced protein diet.

### 3.7 Gene expression

The mRNA expressions of seven digestive enzyme genes in the pancreatic tissue of broilers on d 24 are presented in Table 13. The differences in the expressions of digestive enzyme genes in response to the dietary treatments were observed for *AMY2A* (*P* = 0.011), *CCK* (*P* = 0.031), *CELA1* (*P* = 0.037), and *CELA2A* (*P* = 0.022) genes whereas no responses were observed for *ATP5A*, *CCK1R*, and *PNLIP* genes in the pancreas. The reduction in dietary CP decreased (*P* < 0.05) the expression of the *AMY2A* gene. The addition of either of the four insoluble fibres to the RP diet had no effect (*P* > 0.05) on the expression of *AMY2A* compared to the RP treatment but bagasse, lignocellulose and soy hulls added diets had similar *AMY2A* expression as the NP as well as RP groups. The birds offered a RP diet with bagasse had increased (*P* < 0.05) expression of the *CCK* gene than those offered a RP diet with lignocellulose. The birds offered a RP diet with oat hulls had decreased (*P* < 0.05) expression of the *CELA1* gene compared to those offered a NP diet. The birds offered a RP diet with either lignocellulose or soy hulls had increased (*P* < 0.05) expression of the *CELA2A* gene than those offered a NP diet.

**Table 13:** Expression of genes coding digestive enzymes in pancreatic tissue of broilers offered reduced protein diets with insoluble fibres on d 24^1^.

Treatment	*AMY2A*	*ATP5A*	*CCK1R*	*CCK*	*CELA1*	*CELA2A*	*PNLIP*
Normal CP^2^	1.201^a^	0.935	0.996	0.954^ab^	1.452^a^	0.813^b^	1.023
Reduced CP^3^	0.904^b^	1.031	1.052	1.002^ab^	0.905^ab^	0.996^ab^	0.994
Reduced CP + SB	1.067^ab^	1.125	1.091	1.537^a^	1.152^ab^	1.092^ab^	1.083
Reduced CP + LC	1.046^ab^	0.984	0.924	0.919^b^	0.785^ab^	1.121^a^	1.060
Reduced CP + OH	0.903^b^	0.922	1.047	0.948^ab^	0.745^b^	1.082^ab^	0.897
Reduced CP + SH	0.989^ab^	1.099	1.053	0.954^ab^	0.941^ab^	1.129^a^	0.986
SEM	0.0613	0.0631	0.0851	0.1432	0.1714	0.0701	0.0782
*P*-value	0.011	0.137	0.786	0.031	0.037	0.022	0.662

CP = crude protein; SB = 2% sugarcane bagasse and 1% celite powder; LC = 1% lignocellulose and 2% celite powder; OH = 3% oat hulls; SH = 3% soy hulls; SEM = standard error of the mean.

Within each treatment factor, means in the same column with a different superscript differ significantly (*P* < 0.05), *n* = 8.

1 The relative expression levels of the genes of respective treatment groups are expressed as means of normalized relative quantities (NRQ). Relative quantities for individual genes are scaled to the average across all unknown samples per target gene.

2 Normal protein diet.

3 Reduced protein diet.

The mRNA expressions of digestive enzyme genes in proventricular and jejunal tissues and tight junction protein genes in jejunal tissue of broilers on d 24 are presented in Table 14. The dietary treatments had no effect (*P* > 0.05) on digestive enzyme genes investigated in proventricular tissues, namely, *PepA* and *Pep C*; and jejunal tissues, namely, *APN* and *SI*. Similarly, no responses (*P* > 0.05) to dietary treatments were observed for tight junction protein genes in jejunal tissues, namely, *CLDN1*, *CLDN5*, *JAM2*, *ECADH*, and *TJP1*.

**Table 14:** Expression of genes coding digestive enzymes in proventricular and jejunal tissues and tight junction protein genes in jejunal tissue of broilers offered reduced protein diets with insoluble fibres on d 24^1^.

Treatments	Digestive enzyme genes				Tight junction protein genes				
	APN	SI	Pep A^2^	Pep C^3^	CLDN1	CLDN5	JAM2	ECADH	TJP1
Normal CP^4^	1.180	1.078	1.010	0.850	0.697	0.891	1.100	0.685	1.052
Reduced CP^5^	1.035	1.054	1.138	1.042	0.622	1.008	1.051	1.843	1.105
Reduced CP + SB	0.922	0.859	1.174	1.203	0.715	1.125	1.012	1.764	1.119
Reduced CP + LC	0.995	0.956	0.943	0.977	0.769	1.020	1.138	1.094	1.194
Reduced CP + OH	1.117	1.053	0.969	1.020	1.119	1.113	0.934	1.830	1.079
Reduced CP + SH	1.025	0.898	1.027	1.116	0.789	0.850	1.057	0.704	1.038
SEM	0.1073	0.0751	0.1042	0.0891	0.1171	0.1254	0.1201	0.4942	0.1671
*P-*value	0.611	0.184	0.555	0.127	0.121	0.593	0.879	0.336	0.989

CP = crude protein; SB = 2% sugarcane bagasse and 1% celite powder; LC = 1% lignocellulose and 2% celite powder; OH = 3% oat hulls; SH = 3% soy hulls; SEM = standard error of the mean.

1 The relative expression levels of the genes of respective treatment groups are expressed as means of normalized relative quantities (NRQ). Relative quantities for individual genes are scaled to the average across all unknown samples per target gene.

2 Pepsinogen A gene from proventricular tissue.

3 Pepsinogen C gene from proventricular tissue.

4 Normal protein diet.

5 Reduced protein diet.

The mRNA expressions of two peptide and six AA transporter genes in the jejunal tissue of broilers on d 24 in response to RP diets with insoluble fibres are presented in Table 15. There was no effect (*P* > 0.05) of dietary treatments on peptide transporter genes investigated, namely, *PEPT1* and *PEPT2*; and AA transporter genes, namely, *CAT1*, *ASCT1*, *LAT1*, *EAAT3*, *bo + AT*, and *y + LAT2* in jejunal tissue.

**Table 15:** Expression of genes coding peptide and amino acid transporters in the jejunal tissue of broilers offered reduced protein diets with insoluble fibres on d 24^1^.

Treatments	Peptide transporter genes		Amino acid transporter genes					
	PEPT1	PEPT2	CAT1	ASCT1	LAT1	EAAT3	Bo + AT	Y + LAT2
Normal CP^2^	1.435	1.043	0.787	0.746	0.898	0.127	1.160	0.959
Reduced CP^3^	1.100	0.899	1.341	0.886	1.085	0.123	1.002	0.954
Reduced CP + SB	0.819	0.887	1.200	0.951	1.135	0.999	0.861	0.903
Reduced CP + LC	1.229	0.822	1.085	0.863	1.219	0.855	1.027	0.940
Reduced CP + OH	1.088	1.362	1.071	1.151	1.204	1.129	1.083	0.991
Reduced CP + SH	1.010	0.731	1.170	0.841	0.999	1.036	1.067	0.967
SEM	0.2123	0.3072	0.1751	0.1582	0.1701	0.1133	0.1202	0.0521
*P*-value	0.458	0.792	0.362	0.591	0.752	0.483	0.627	0.891

CP = crude protein; SB = 2% sugarcane bagasse and 1% celite powder; LC = 1% lignocellulose and 2% celite powder; OH = 3% oat hulls; SH = 3% soy hulls; SEM = standard error of the mean.

1 The relative expression levels of the genes of respective treatment groups are expressed as means of normalized relative quantities (NRQ). Relative quantities for individual genes are scaled to the average across all unknown samples per target gene.

2 Normal protein diet.

3 Reduced protein diet.

The mRNA expressions of two glucose and two free fatty acid transporter genes in the jejunal tissue of broilers on d 24 in response to RP diets with insoluble fibres are presented in Table 16. There was no effect (*P* > 0.05) of dietary treatments on glucose transporter genes, namely, *GLUT2* and *GLUT5*; and free fatty acid transporter genes, namely, *FFAR2* and *FFAR4* in jejunal tissue.

**Table 16:** Expression of genes coding glucose and free fatty acid transporters in jejunal tissue of broilers offered reduced protein diets with insoluble fibres on d 24^1^.

Treatments	Glucose transporter genes		Free fatty acid transporter genes	
	*GLUT2*	*GLUT5*	*FFAR2*	*FFAR4*
Normal CP^2^	1.003	0.848	0.618	1.165
Reduced CP^3^	1.081	0.98	0.907	1.012
Reduced CP + SB	1.056	0.813	1.073	0.951
Reduced CP + LC	0.949	0.855	0.960	1.100
Reduced CP + OH	1.081	0.871	1.672	1.128
Reduced CP + SH	0.931	0.786	0.901	0.935
SEM	0.1142	0.1153	0.2971	0.1152
*P-*value	0.896	0.939	0.245	0.623

CP = crude protein; SB = 2% sugarcane bagasse and 1% celite powder; LC = 1% lignocellulose and 2% celite powder; OH = 3% oat hulls; SH = 3% soy hulls; SEM = standard error of the mean.

1 The relative expression levels of the genes of respective treatment groups are expressed as means of normalized relative quantities (NRQ). Relative quantities for individual genes are scaled to the average across all unknown samples per target gene.

2 Normal protein diet.

3 Reduced protein diet.

## 4 Discussion

The reduction in dietary CP from 217 to 197 g/kg in grower and from 198 to 178 g/kg in finisher wheat-based diets compromised the growth performance of broilers as demonstrated by a 3.3% drop in FI, 4.7% decrease in WG and 2.1 points greater FCR during 10 to 35 d. While compromised FCR and decreased WG in RP wheat-based diets are reported in many studies (Macelline et al., 2023,b, c; Selle et al., 2022), the reductions in FI as observed in this study were reported in only a few other studies (Greenhalgh et al., 2020). For instance, Macelline et al. (2023,b, c), Chrystal et al. (2021), and Yin et al. (2020) reported no change in FI of broilers when dietary CP was moderately reduced in the range of 20 to 30 g/kg from the normal levels in wheat-based diets. Although the RP diet in the current study contained only 20 g/kg lower CP than the NP treatment, it may still be possible that some of the supplemental free AA in the RP diet were catabolised in the gut mucosa to produce ammonia triggering depressed FI (Chrystal et al., 2021). In contrast, Lambert et al. (2023) displayed no effect on growth performance in broilers fed wheat-based diets when dietary CP was reduced by 20 g/kg during d 10 to 30. However, it should be noted that their study used supplemental L-Leu and L-His in reduced CP treatments which were not used in the current study. Overall, there is a consensus among different studies that a marked reduction in dietary CP (>30 g/kg) depresses FI in broilers (Chrystal et al., 2021; Macelline et al., 2023a,b, c), but the discrepancy in FI at moderately reduced CP wheat-based diets should be further investigated as this level of dietary CP reduction is practical for use in the poultry industry at present. It should be noted that all the diets in this experiment were supplemented with xylanase and phytase enzymes to counteract any anti-nutritive effects of soluble NSP or phytate content in the feed that may affect the growth performance of broilers.

Insoluble fibres were used in the current study as a nutritional strategy to alleviate performance loss associated with wheat-based RP feeding program as the benefits of insoluble fibres on growth performance and protein digestibility in broilers are well documented (Mateos et al., 2012; Jimenez-Moreno et al., 2013; Kheravii et al., 2017). In the current study, adding insoluble fibres to RP diets did not affect FI except for soy hulls which increased FI of birds and pushed it closer to the NP treatment. In contrast, Kheravii et al. (2017) reported increased FI in broilers when bagasse was added at 2% in diets. This discrepancy may be due to the fact that in the current study all the insoluble fibres were formulated into the diets to achieve the same dietary AME level across all the treatments, whereas, in the study of Kheravii et al. (2017), bagasse was added on top of the complete feed which diluted the nutrient density of the diets and possibly resulted in higher FI. During diet formulation, CP contributions from insoluble fibres were not taken into consideration in the current study but with soy hulls contributing around 0.3% intact protein into the feed (considering approx. 10% CP and 3.0% inclusion of soy hulls), the analysed dietary CP in RP + soy hulls treatment was 0.3% points higher than the calculated value (for eg. in analysed grower feed) which pushed dietary CP of RP + soy hulls treatment closer to the NP treatment. In future studies, CP contribution from soy hulls should be taken into consideration during diet formulation to see any impact.

Reduction in dietary CP decreased WI by 11.45% and WI:FI from 2.052 to 1.875 during d 10 to 35. These findings are in accord with the findings of Lambert et al. (2023) who reported a linear reduction in WI and WI:FI of broilers when dietary CP was reduced by 10 to 30 g/kg in wheat-based diets. A lower WI reflects a lower intake of nitrogen in the RP diet as there is a reduced need of water to excrete surplus nitrogen (Lemme et al., 2019). A lower intake of water complements other nutritional and managemental factors to manage the risk of wet litter in broiler production. Adding either of the four insoluble fibres in moderate amounts to the RP diet did not affect WI and WI:FI in the current study which are favourable outcomes for the poultry industry. In fact, bagasse, soy hulls, and lignocellulose have 3 to 5 times more water holding capacity than cereal grains and may prevent a sudden release of water in the hindgut to prevent wet litter (Cadogan et al., 2024). The addition of moderate amounts of insoluble fibres, which have little effect on intestinal viscosity (Bach Knudsen, 2001) may complement a wheat-based RP feeding program to further improve litter quality beyond what is achieved by the RP diet alone. However, this hypothesis, together with the effect of different ratios of insoluble to soluble NSP in RP diets on growth performance and litter quality, needs to be investigated in the future.

The addition of soy hulls and bagasse in RP diets increased the WG of birds by 73 and 37 g during d 10 to 35, respectively, and improved feed efficiencies, making them similar to the NP treatment. Bagasse had only 1.0% analysed CP which contributes a negligible CP to RP diets at a 30 g/kg inclusion level. The improvements in feed efficiency with the addition of bagasse in RP diets can be linked to their particle size and improved gizzard function which helps in the regulation of the flow of nutrients through the GI tract (Mateos et al., 2012; Svihus, 2011). In the current study, the addition of bagasse and oat hulls in RP diets increased the relative weight of gizzard both on d 24 and 35. For instance, on d 35, bagasse and oat hulls in RP diets increased the relative weight of gizzard by 8.7% and 17.3% respectively, compared to the RP control. The increase in gizzard weight has been linked to the accumulation of coarser particles that provoke the grinding activity, thereby resulting in increased size of the gizzard muscles (Svihus, 2011). If the increase in relative gizzard weight could improve the gizzard function, nutrient digestibility, and growth performance in broilers as suggested previously (Jha and Mishra, 2021; Mateos et al., 2012), higher performance benefits would be expected with the RP + oat hulls treatment in the current study as this treatment had the highest relative gizzard weight, but there was no response on growth performance. Studies have found that WG, FCR, and AID of starch were improved in broilers when oat hulls were included at 30 g/kg in the NP diet (Barekatain et al., 2017; Jimenez-Moreno et al., 2013; Gonzalez-Alvarado et al., 2010). The discrepancy in response to oat hulls addition on growth performance but relative gizzard weight between their studies and our study suggests that dietary CP and starch levels and ingredient and nutrient composition of the diets dictate growth performance response when insoluble fibres are included in the diets. A NP diet contains more intact protein which results in a more indigestible protein fraction in the gut for insoluble fibres to operate, unlike RP diets where a high proportion of intact proteins are replaced with free AA making less room for additional CP digestion. It should also be noted that oat hulls may contain a significant amount of intact starch during the dehulling process, which was also observed in the current study, which is not desirable in RP diets that already have a high level of starch.

Improved relative gizzard weight, however, translated to higher WG and improved feed efficiency when bagasse was added to the RP diet although nutrient (starch and protein) digestibility was not improved. In fact, the addition of bagasse or soy hulls in the RP diet decreased the AID coefficient of starch but had no effect on the AID coefficient of CP in the distal ileum which supported the growth performance of birds. In this experiment, the impaired performance of birds fed a moderately reduced CP diet may be due to: a) increased (15% to 17%) digestible starch coming from wheat which expanded dietary starch to CP ratio by 0.49 to 0.58; b) lower FI, and c) increased AID coefficient of starch in distal ileum. These three parameters may have increased the apparent starch disappearance rate and starch to CP disappearance rate ratio in the small intestine affecting starch and protein digestive dynamics (Selle and Liu, 2019) and broiler growth performance.

Starch digestibility is inversely related to FI in broilers fed a wheat-based pelleted diet (Svihus, 2011) which supports our finding of a high AID coefficient of starch in distal ileum concomitant with lower FI in birds fed a RP diet. In another study, ileal starch digestibility was improved with increasing dietary starch levels (Itani et al., 2020) which corroborates our findings as RP diets contained 15% to 17% higher starch than the NP diet. In contrast, no effect of dietary CP reduction on the AID coefficient of starch in distal ileum was observed by others (Macelline et al., 2023a). In fact, Macelline et al. (2023b) reported a decreased AID coefficient of starch in the distal ileum when dietary CP was reduced from 210 to 180 g/kg in wheat-based diets. It is, however, worth noting that a very high AID coefficient of starch in distal ileum ranging between 0.980 and 0.998 has been consistently reported in recent wheat-based feeding studies (Macelline et al., 2023a,b, c) regardless of dietary CP levels which are contrary to our findings of AID coefficient of 0.936 with a NP diet and a slightly higher coefficient of 0.962 with a RP diet, and the findings of Chrystal et al. (2021) who reported even lower AID coefficient of starch in the distal ileum, such as a coefficient of 0.887 with a 222 g/kg CP diet and a coefficient of 0.854 with a 193 g/kg CP diet. Unless high gelatinisation of starch achieved through longer conditioning time and higher temperature during steam pelleting process (compared to cold pelleting at 65 °C) increased AID of starch close to 1.000, such high levels of ileal starch digestibility consistently reported in above studies need to be investigated as this discrepancy in starch digestibility among these studies at various segments of the small intestine may dictate growth performance response of broilers when dietary CP is reduced. Several other studies that used wheat-based pelleted feed with xylanase reported a lower AID of starch in the ileum of broilers and are similar to our study (Itani et al., 2020; Svihus, 2011, 2014). The impact of the degree of starch gelatinisation in pelleted feed on starch digestibility in broilers has not been thoroughly investigated, although it is well accepted that the increase in starch gelatinisation will increase the pellet quality. In the current study, the addition of insoluble fibres in the RP diet, regardless of the source, decreased the starch content in feed by 9.3% to 14.6% and dietary starch to CP ratio by 0.18 to 0.36 bringing these parameters closer to the NP treatment. This was achieved through the addition of canola oil (fat) as a source of energy at the expense of wheat in feed to compensate for the 30 g/kg fibre additions so as to get the same AME level in all the dietary treatments. As a result, total dietary fat in the fibre treatments was slightly higher than the RP control diet but still at or below 6.0% which would not affect the pellet quality.

The addition of bagasse or soy hulls in RP diets reduced the AID coefficient of starch in the distal ileum of broilers and made them similar to the NP treatment. This together with lower dietary starch (as described above) may have reduced the starch disappearance rate and brought them closer to the NP treatment. All these effects partially attenuated the compromised growth performance of broilers fed RP diet possibly by allowing better AA uptakes relative to glucose in the small intestine. In a recent study, Ghimire et al. (2024) reported that some coarser oat hulls that were added to broiler diets escaped grinding in the gizzard and passed further down the digestive tract. This finding challenges the previous assumption that particles are ground to a certain size in the gizzard before they pass down to the small intestine (Svihus, 2011). It is plausible that larger bagasse and soy hull particles in the current study escaped grinding in the gizzard, passed down into the small intestine, and incapsulated some of the starch in the gut preventing their exposure to the surrounding moisture and digestive enzymes resulting in decreased starch digestibility and disappearance rate. Nutritional strategies that may decrease starch digestibility and disappearance rate in the intestine are therefore deemed beneficial in wheat-based RP feeding program for broilers.

The expression of digestive enzyme genes in pancreatic tissue offers insights into the digestion of nutrients in broilers. The reduction in dietary CP decreased the expression of the *AMY2A* gene thus affecting the starch digestibility in the current study. Amylase is secreted endogenously by saliva and pancreas, and helps in the digestion of starch (Moran, 1985). The lower *AMY2A* gene expression in the pancreas may be a reflection of an increased level of dietary starch resulting in higher AID of starch in the distal ileum of broilers fed a RP diet. Although the addition of either of the four insoluble fibres to the RP diet had no effect on the expression of *AMY2A* compared to the RP treatment, bagasse, and soy hulls added diets had similar *AMY2A* expressions as the NP group as well as RP groups indicating a shift towards NP. This complements the responses observed in apparent starch digestibility coefficient and disappearance rate with the addition of these fibres in this study. Kheravii et al. (2018) reported greater expression of pancreatic *AMY2A* and *CELA1* genes in broilers fed a diet supplemented with 2% bagasse which partially corroborates our finding although we did not see the same response on *CELA1* gene expression when bagasse was added to RP diet and fed to birds. The reduction in dietary CP had no effect on AID coefficients of CP in the distal jejunum or ileum and the addition of either of these insoluble fibres had no effect on AID coefficients of CP in the distal ileum. However, the AID of Met and Gly was increased when the dietary CP was reduced but the addition of either of the four insoluble fibre sources in the RP diet had no further effect on the AID of AA.

The gene expression study and microbiota composition provide insight into the intestinal health and physiology of animals. It has been reported that severe reduction of dietary CP compromises intestinal health parameters in broilers such as mRNA expression of tight junction proteins, intestinal permeability, and caecal microbiota composition (Barekatain et al., 2023). In the current study, the lack of response of dietary treatments on tight junction protein genes in jejunal tissues, namely, *CLDN1*, *CLDN5*, *JAM2*, *ECADH*, and *TJP1* suggests that a moderate (20 g/kg) reduction in dietary CP and a moderate (30 g/kg) inclusion of insoluble fibre in the diets may not affect the intestinal health of broilers. Although the reduction in dietary CP had no effect on bacterial load in the intestinal contents, the addition of insoluble fibre to RP diets had some minor effects on selected bacterial population in the ileal and caecal contents of broilers. For instance, the birds offered a RP diet with oat hulls had lower *Lactobacillus* counts in the ileal contents compared to those offered a RP diet with lignocellulose or soy hulls. The birds offered a RP diet with either oat hulls or bagasse had the lowest counts of total bacteria in the ileal contents compared to others. It has been reported that a higher dietary soluble NSP increases ileal viscosity and *Lactobacillus* counts in ileal digesta of broilers (Nguyen et al., 2021) and it is likely that increased ileal viscosity may increase total bacteria counts in the ileum where *Lactobacilli* are dominant (Selle et al., 2022). The addition of bagasse or oat hulls in RP diets increased dietary canola oil (fat) at the expense of wheat to achieve the same AME level as the RP treatment. It is plausible that decreased wheat and increased fibre additions may have increased the insoluble to soluble NSP ratio and lowered intestinal viscosity thus lowering *Lactobacillus* and total bacteria counts in ileal contents. Similarly, the birds offered a RP diet with either bagasse or oat hulls had lower *Lactobacillus* counts in the caecal contents compared to those offered a RP diet and a RP diet with soy hulls. As discussed, insoluble fibres which have negligible intact protein or poor fermentation characteristics may lower the total bacterial load in the ileum and *Lactobacillus* counts in the ileal and caecal contents.

## 5 Conclusion

The findings from this study suggest that the reduction in dietary CP at 20 g/kg or below in a wheat-based diet will negatively affect the performance of broilers characterized by lower FI, lower WG, greater FCR, and higher abdominal fat pad. Insoluble fibre may be used as a dietary ingredient to provide benefits to a RP feeding program, but the response depends on the source of fibre used as only bagasse and soy hulls supplementation partially attenuated compromised growth performance resulting from the reduction in dietary CP. The beneficial effects of these selected insoluble fibres in RP diets are likely due to reduced dietary starch, reduced dietary starch to protein ratio, and increased structural components in feed translating to increased relative weight of gizzard which effectively regulated the flow of nutrients in the gut, decreased starch digestibility in distal ileum which possibly led to reduced starch disappearance rate and starch to protein disappearance rate ratio. Further researches are required to examine the effect of different inclusion levels and particle size distribution of insoluble fibres on growth performance and litter quality of broilers fed both wheat and maize-based reduced CP diets.

## Credit Author Statement

**Nishchal K. Sharma:** Writing – review & editing, Writing – original draft, Project administration, Methodology, Investigation, Formal analysis, Data curation. **Sarbast K. Kheravii:** Writing – review & editing, Methodology, Data curation. **Mingan Choct:** Writing – review & editing, Conceptualization. **Karen Gurney:** Writing – review & editing, Methodology. **Shu-Biao Wu:** Writing – review & editing, Supervision, Project administration, Methodology, Funding acquisition, Conceptualization.

## Declaration of competing interest

We declare that we have no financial and personal relationships with other people or organizations that can inappropriately influence our work, and there is no professional or other personal interest of any nature or kind in any product, service and/or company that could be construed as influencing the content of this paper.

## References

[bib1] AOAC (1990).

[bib2] Aviagen Ross 308 (2014).

[bib3] Aviagen Ross 308 (2019).

[bib4] Boogers I., Plugge W., Stokkermans Y.Q., Duchateau A.L. (2008). Ultra-performance liquid chromatographic analysis of amino acids in protein hydrolysates using an automated pre-column derivatisation method. J Chromatogr A.

[bib5] Bach Knudsen K.E. (2001). The nutritional significance of “dietary fibre” analysis. Anim Feed Sci Technol.

[bib6] Barekatain R., Swick R.A., Toghyani M., de Koning C.T. (2017). Interactions of full-fat canola seed, oat hulls as an insoluble fiber source and pellet temperature for nutrient utilization and growth performance of broiler chickens. Poult Sci.

[bib7] Barekatain R., Chrystal P.V., Nowland T., Moss A.F., Howarth G.S., Van T.T.H., Moore R.J. (2023). Negative consequences of reduced protein diets supplemented with synthetic amino acids for performance, intestinal barrier function, and caecal microbiota composition of broiler chickens. Anim Nutr.

[bib8] Cadogan D.J., Choct M. (2024). Importance of fibre and fibre characteristics in modulating feed intake in broiler breeders. Proc Aus Poult Sci Symp.

[bib9] Chrystal P.V., Greenhalgh S., McInerney B.V., McQuade L.R., Akter Y., Cesar de Paula Dorigam J., Selle P.H., Liu S.Y. (2021). Maize-based diets are more conducive to crude protein reductions than wheat-based diets for broiler chickens. Anim Feed Sci Technol.

[bib10] Cowieson A.J., Zaefarian F., Knap I., Ravindran V. (2017). Interactive effects of dietary protein concentration, a mono-component exogenous protease and ascorbic acid on broiler performance, nutritional status and gut health. Anim Prod Sci.

[bib11] Dean D.W., Bidner T.D., Southern L.L. (2006). Glycine supplementation to low protein, amino acid-supplemented diets supports optimal performance of broiler chicks. Poult Sci.

[bib12] Englyst H.N., Quigley M.E., Hudson G.J. (1994). Determination of dietary fibre as non-starch polysaccharides with gas–liquid chromatographic, high-performance liquid chromatographic or spectrophotometric measurement of constituent sugars. Analyst.

[bib13] Ghimire S., Itani K., Kaczmarek S., Smith A., Svihus B. (2024). Influence of particle size and inclusion level of oat hulls on retention and passage in the anterior digestive tract of broilers. Br Poult Sci.

[bib14] González-Alvarado J.M., Jiménez-Moreno E., Lázaro R., Mateos G.G. (2007). Effect of type of cereal, heat processing of the cereal, and inclusion of fiber in the diet on productive performance and digestive traits of broilers. Poult Sci.

[bib15] González-Alvarado J.M., Jiménez-Moreno E., González-Sánchez D., Lázaro R., Mateos G.G. (2010). Effect of inclusion of oat hulls and sugar beet pulp in the diet on productive performance and digestive traits of broilers from 1 to 42 days of age. Anim Feed Sci Technol.

[bib16] Greenhalgh S., McInerney B.V., McQuade L.R., Chrystal P.V., Khoddami A., Zhuang M.A.M., Liu S.Y., Selle P.H. (2020). Capping dietary starch:protein ratios in moderately reduced crude protein, wheat-based diets showed promise but further reductions generated inferior growth performance in broiler chickens. Anim Nutr.

[bib17] Itani K., Granstad S., Kaldhusdal M., Mydland L.T., Svihus B. (2020). Varying starch to fat ratios in pelleted diets: I. Effects on nutrient digestibility and production performance in Eimeria–challenged broiler chickens. Br Poult Sci.

[bib18] Jha R., Mishra P. (2021). Dietary fiber in poultry nutrition and their effects on nutrient utilization, performance, gut health, and on the environment: a review. J Anim Sci Biotechnol.

[bib19] Jiménez-Moreno E., Frikha M., de Coca-Sinova A., Garcia J., Mateos G.G. (2013). Oat hulls and sugar beet pulp in diets for broilers 1. Effects on growth performance and nutrient digestibility. Anim Feed Sci Technol.

[bib20] Kheravii S.K., Swick R.A., Choct M., Wu S.-B. (2017). Dietary sugarcane bagasse and coarse particle size of corn are beneficial to performance and gizzard development in broilers fed normal and high sodium diets. Poult Sci.

[bib21] Kheravii S.K., Swick R.A., Choct M., Wu S.-B. (2018). Upregulation of genes encoding digestive enzymes and nutrient transporters in the digestive system of broiler chickens by dietary supplementation of fiber and inclusion of coarse particle size corn. BMC Genom.

[bib22] Lambert W., Berrocoso J.D., Swart B., van Tol M., Bruininx E., Willems E. (2023). Reducing dietary crude protein in broiler diets positively affects litter quality without compromising growth performance whereas a reduction in dietary electrolyte balance further improves litter quality but worsens feed efficiency. Anim Feed Sci Technol.

[bib23] Lemme A., Hiller P., Klahsen M., Taube V., Stegemann J., Simon I. (2019). Reduction of dietary protein in broiler diets not only reduces n-emissions but is also accompanied by several further benefits. J Appl Poultry Res.

[bib24] Macelline S.P., Chrystal P.V., Inanan C., Toghyani M., Selle P.H., Liu S.Y. (2023). The influence of dietary crude protein concentrations, grain types and arginine:lysine ratios on the performance of broiler chickens. Anim Nutr.

[bib25] Macelline S.P., Kidd M.T., Chrystal P.V., Toghyani M., Selle P.H., Liu S.Y. (2023). The influence of non-bound amino acid inclusions and starch-protein digestive dynamics on growth performance of broiler chickens offered wheat-based diets with two crude protein concentrations. Anim Nutr.

[bib26] Macelline S.P., Chrystal P.V., Toghyani M., Selle P.H., Liu S.Y. (2023). Dietary crude protein reductions in wheat-based diets with two energy densities compromised performance of broiler chickens from 15 to 36 days post-hatch. Poult Sci.

[bib27] Mateos G.G., Jiménez-Moreno E., Serrano M.P., Lázaro R.P. (2012). Poultry response to high levels of dietary fiber sources varying in physical and chemical characteristics. J Appl Poultry Res.

[bib28] Moran E.T. (1985). Digestion and absorption of carbohydrates in fowl and events through perinatal development. J Nutr.

[bib29] Morgan N.K., Keerqin C., Wallace A., Wu S.-B., Choct M. (2019). Effect of arabinoxylo-oligosaccharides and arabinoxylans on net energy and nutrient utilization in broilers. Anim Nutr.

[bib30] Nguyen H.T., Bedford M.R., Wu S.-B., Morgan N.K. (2021). Soluble non-starch polysaccharide modulates broiler gastrointestinal tract environment. Poult Sci.

[bib31] NHMRC (2013).

[bib32] Selle P.H., Liu S.Y. (2019). The relevance of starch and protein digestive dynamics in poultry. J Appl Poultry Res.

[bib33] Selle P.H., Macelline S.P., Greenhalgh S., Chrystal P.V., Liu S.Y. (2022). Identifying the shortfalls of crude protein-reduced, wheat-based broiler diets. Anim Nutr.

[bib34] Selle P.H., Macelline S.P., Toghyani M., Liu S.Y. (2024). The potential of glutamine supplementation in reduced-crude protein diets for chicken-meat production. Anim Nutr.

[bib35] Sharma N.K., Choct M., Dunlop M.W., Wu S.B., Castada H.Z., Swick R.A. (2017). Characterisation and quantification of changes in odorants from litter headspace of meat chickens fed diets varying in protein levels and additives. Poult Sci.

[bib36] Short F.J., Gorton P., Wiseman J., Boorman K.N. (1996). Determination of titanium dioxide added as an inert marker in chicken digestibility studies. Anim Feed Sci Technol.

[bib37] Svihus B. (2011). The gizzard: function, influence of diet structure and effects on nutrient availability. Worlds Poult Sci J.

[bib38] Svihus B. (2014). Starch digestion capacity of poultry. Poult Sci.

[bib39] Theander O., Westerlund E.A. (1986). Studies on dietary fiber. 3. Improved procedures for analysis of dietary fiber. J Agric Food Chem.

[bib40] Theander O., Aman P., Westerlund E., Andersson R., Pettersson D. (1995). Total dietary fiber determined as neutral sugar residues, uronic acid residues, and Klason lignin (the Uppsala method): collaborative study. J AOAC Int.

[bib41] Yin D., Chrystal P.V., Moss A.F., Liu S.Y., Yuan J., Selle P.H. (2020). Effects of reducing dietary crude protein and whole grain feeding on performance and amino acid metabolism in broiler chickens offered wheat-based diets. Anim Feed Sci Technol.

