# Supplementary material for: Supplementation of specific insoluble fibres partially attenuates compromised growth performance of broilers fed wheat-based reduced crude protein diets
Source: Anim Nutr. 2025 Aug 26;23:479–92. doi: 10.1016/j.aninu.2025.06.009 (PMC12664108; doi:10.1016/j.aninu.2025.06.009)
Supplement: Multimedia component 1 [file mmc1.docx]

**Table S1**

Sequences of primers used for the qPCR analysis of selected genes in pancreatic and jejunal tissues of 24 d old broilers.

| Genes | Gene full names | Primer sequences (5ʹ-3ʹ) | Ta, ^o^C | Amplicon size, bp | Reference |  |  |
| --- | --- | --- | --- | --- | --- | --- | --- |
| **Digestive enzymes, peptide and AA transporter genes** | | | | | |  |  |
| *AMY2A* | Pancreatic alpha 2A amylase | F-CGGAGTG↓GATGTTAACGACTGG  R-ATGTTCGCAGACCCAGTCATTG | 60 | 112 | Kheravii et al. (2018) |  |  |
| *APN* | Aminopeptidase N | F-AATACGCGCTCGAGAAAACC  R-AGCGGGTACGCCGTGTT | 60 | 70 | Gilbert et al. (2007) |  |  |
| *ASCT1* | Alanine, serine, Cysteine, and threonine transporter (SLC1A4) | F-TTGGCCGGGAAGGAGAAG  R-AGACCATAGTTGCCTCATTGAATG | 60 | 63 | Paris et al. (2013) |  |  |
| *ATP5A1W* | ATP synthase subunit alpha | F-GGCAATGAAACAGGTGGCAG  R-GGGCTCCAGCTTGTCTAAGTGA | 60 | 232 | Nafari et al. (2021) |  |  |
| *B^o,+^AT* | Solute carrier family 7, member 9 | F-CAGTAGTGAATTCTCTGAGTGTGAAGCT  R- GCAATGATTGCCACAACTACCA | 60 | 88 | Gilbert et al. (2007) |  |  |
| *CAT1* | Cationic amino acid transporter-1 | F-CAAGAGGAAAACTCCAGTAATTGCA  R- AAGTCGAAGAGGAAGGCCATAA | 60 | 75 | Gilbert et al. (2007) |  |  |
| *CCK* | Cholecystokinin | F-AGGTTCCACTGGGAGGTTCT  R-CGCCTGCTGTTCTTTAGGAG | 60 | 152 | Kheravii et al. (2018) |  |  |
| *CCK1R* | Cholecystokikin type 1 receptor | F-CACTTACTTCATGGGTATCTCTGTG  R-GATGGCAACAAGGTTGAATGTAGA | 60 | 55 | Ohkubo et al. (2007) |  |  |
| *CELA1* | Chymotrypsin-like elastase family, member 1 | F-AGCGTAAGGAAATGGGGTGG  R-GTGGAGACCCCATGCAAGTC | 60 | 75 | Kheravii et al. (2018) |  |  |
| *CELA2A* | Chymotrypsin-like elastase family member 2A | F-GAGGGGAAGATGCAAGACCAT  R-CCTTGCTCCTCAGCTTCTAGG | 60 | 196 | Kheravii et al. (2018) |  |  |
| *EEAT3* | Excitatory amino acid transporter 3 | F-TGCTGCTTTGGATTCCAGTGT  R-AGCAATGACTGTAGTGCAGAAGTAATATATG | 60 | 79 | Su et al. (2014) |  |  |
| *FFAR2* | Free fatty acid receptor 2 | F-GCTCGACCCCTTCATCTTCT  R-ACACATTGTGCCCCGAATTG | - | - | Slawinska et al. (2019) |  |  |
| *FFAR4* | Free fatty acid receptor 4 | F-AGTGTCACTGGTGAGGAGATT  R-ACAGCAACAGCATAGGTCAC | - | - | Slawinska et al. (2019) |  |  |
| *GLUT2* | Glucose transporter-2 (SLC2A2) | F-TGATCGTGGCACTGATGGTT  R-CCACCAGGAAGAC↓GGAGATA | - | - | Slawinska et al. (2019) |  |  |
| *GLUT5* | Glucose transporter-5 | F-ACGGTTCCCAGAGCAAGTTA R-GTCTTGCATGTATGGGGCTG | - | - | Slawinska et al. (2019) |  |  |
| *LAT1* | L type amino acid transporter-1 (SLC7A5) | F-GATTGCAACGGGTGATGTGA  R-CCCCACACCCACTTTTGTTT | 60 | 70 | Gilbert et al. (2007) |  |  |
| *PepT1* | Peptide transporter-1 | F-TACGCATACTGTCACCATCA  R-TCCTGAGAACGGACTGTAAT | 60 | 205 | Guo et al. (2014) |  |  |
| *PepT2* | Peptide transporter-2 | F-TGACTGGGCATCGGAACAA  R-ACCCGTGTCACCATTTTAACCT | 60 | 63 | Paris and Wong (2013) |  |  |
| *PNLIP* | Pancreatic lipase | F-GCATCTGGGAAG↓GAACTAGGG  R-TGAACCACAAGCATAGCCCA | 60 | 113 | Kheravii et al. (2018) |  |  |
| *SI* | Sucrose isomaltase | F-CTTTAAG^↓^ATGGGCAAGAGGAAG  R-CCACCACCAGGCAAAAGAGG | 60 | 65 | Kheravii et al. (2018) |  |  |
| *Y+LAT2* | Y+ L amino acid transporter-2 | F-GCCCTGTCAGTAAATCAGACAAGA  R-TTCAGTTGCATTGTGTTTTGGTT | 60 | 82 | Gilbert et al. (2007) |  |  |
| **Tight junction proteins and inflammatory-related genes** | | | | | |  |  |
| *CLDN1* | Claudin 1 | F-CTTCATCATTGCAGGTCTGTCAG  R-AAATCTGGTGTTAACGGGTGTG | 60 | 103 | Zanu et al. (2020) |  |  |
| *CLDN5* | Claudin 5 | F-GCAGGTCGCCAGAGATACAG  R-CCACGAAGCCTCTCATAGCC | 61 | 162 | Zanu et al. (2020) |  |  |
| *JAM2* | Junctional adhesion 2 | F-AGACAGGAACAGGCAGTGCTAG  R-ATCCAATCCCATTTGAGGCTAC | 60 | 135 | Zanu et al. (2020) |  |  |
| *TJP1* | Tight junction protein | F-GGATGTTTATTTGGGCGGC  R-GTCACCGTGTGTTGTTCCCAT | 60 | 187 | Zanu et al. (2020) |  |  |
| **Housekeeping genes** | | | | | |  |  |
| *HMBS* | Hydroxymethylbilane synthase | F-GGCTGGGAGAATCGCATAGG R-TCCTGCAGGGCAGATACCAT | - | 131 | Yin et al. (2011) |  |  |
| *HPRT1* | Hypoxanthine guanine  phosphoribosyl transferase 1 | F-ACTGGCTGCTTCTTGTG  R-GGTTGGGTTGTGCTGTT | 63 | 245 | Yang et al. (2013) |  |  |
| *TBP* | TATA-Box binding protein | F-TAGCCCGATGATGCCGTAT  R-GTTCCCTGTGTCGCTTGC | 62 | 147 | Li et al. (2005) |  |  |
